# Vitamin D treatment attenuates 2,4,6-trinitrobenzene sulphonic acid (TNBS)-induced colitis but not oxazolone-induced colitis

**DOI:** 10.1038/srep32889

**Published:** 2016-09-13

**Authors:** Tianjing Liu, Yongyan Shi, Jie Du, Xin Ge, Xu Teng, Lu Liu, Enbo Wang, Qun Zhao

**Affiliations:** 1 Department of Pediatric Orthopedics, Shengjing Hospital of China Medical University, NO. 36 Sanhao Street, Shenyang, Liaoning 110004, P. R. China; 2 Key Laboratory of Health Ministry for Congenital Malformation, Shengjing Hospital of China Medical University, NO. 36 Sanhao Street, Shenyang, Liaoning 110004, P. R. China; 3 Department of Neonatology, Shengjing Hospital of China Medical University, NO. 36 Sanhao Street, Shenyang, Liaoning 110004, P. R. China; 4 Institute of Metabolic Disease Research and Drug Development, China Medical University, 77 Puhe Road, Shenyang, Liaoning 110004, P. R. China; 5 Department of Gastroenterology, Shengjing Hospital of China Medical University, NO. 36 Sanhao Street, Shenyang, Liaoning 110004, P. R. China

## Abstract

Crohn’s disease (CD) and ulcerative colitis (UC) have different immunological mechanisms, while both of them are potential targets of vitamin D treatment. In this study, we have tried to address the role of vitamin D in CD and UC using two mouse models. Mice of C57B6L were given vitamin D before the induction of colitis. Our results showed that vitamin D attenuated 2,4,6-trinitrobenzene sulphonic acid (TNBS)-induced colitis but not oxazolone-induced colitis. Vitamin D could preserve the local histology, alleviate inflammation, suppress apoptosis, maintain tight junction function and decrease permeability. Interestingly, it has more of an effect on local structure preservation and inflammation inhibition in CD than in UC mice. Vitamin D blocked the increase of helper T-cell type 1 (Th1)- and helper T-cell type 17 (Th17)-related cytokines in TNBS-induced colitis. But the increase of helper T-cell type 2 (Th2)- and regulatory T cells (Treg)-related cytokines was augmented at the same time in oxazolone-induced colitis which counteracted each other. Our study helps elucidate the differential protective effects of vitamin D on CD and UC patients, as reported in literature.

Crohn’s disease (CD) and ulcerative colitis (UC) are the two main types of chronic inflammatory bowel diseases (IBDs). Despite the obscurity concerning the pathogenesis of IBD, increasing evidence has indicated that it is caused by an inappropriate inflammatory response to intestinal microbes in a genetically susceptible host^1^. CD has been associated with the induction of helper T-cell type 1 (Th1) and helper T-cell type 17 (Th17) cells. T cells isolated from intestinal lesions in CD patients produce excessive IFN-γ (the major cytokine in Th1-mediated inflammation) but produce decreased levels of IL-4 [the major cytokine in helper T-cell type 2 (Th2)-mediated inflammation]^2^. Another study has demonstrated that antigen-presenting cells from the lamina propria of CD patients produce IL-12, a cytokine that could direct the differentiation of Th1-producing effector T cells^3^. More recently, the IL-23/IL-17 axis has been implicated in chronic intestinal inflammation^4^, possibly by inducing Th17 cells to secrete IL-17, IL-6, and TNF-α^5^.

The immunopathogenesis of UC is quite different from that of CD in that there is no excessive IFN-γ in UC colonic tissue. The excessive Th1 cell response in CD is characterized by increased IL-12, IFN-γ, and TNF-α production, while in UC it skews towards a Th2 cell response via increased IL-4 and/or IL-13 production. Increased IL-5 secretion has also been found in UC tissue^2^. Compared with normal and CD tissue, lamina propria cells isolated from UC tissue produce more IL-13 after stimulation^6^. IL-13 has recently been identified as an important effector cytokine that impairs epithelial barrier function by affecting epithelial apoptosis, tight junctions, and restitution velocity^7^. Because IL-5 and IL-13 are both Th2 cytokines, UC appears to be more of a Th2-mediated inflammatory disease.

1,25-dihydroxyvitamin D_3_, when bound to the vitamin D receptor (VDR), plays a pivotal role in the immunoregulatory process, as demonstrated in systemic lupus erythematosus, experimental allergic encephalomyelitis and autoimmune diabetes mellitus^8^,^9^. Vitamin D (VD) deficiency highly correlates with the incidence of IBD, especially CD^10^,^11^,^12^,^13^. A recent meta-analysis suggested that an increased risk of IBD is associated with several polymorphisms of the VDR gene^14^. Over the past 20 years, murine models of intestinal mucosal inflammation have been widely utilized to study IBD. 2,4,6-trinitrobenzene sulphonic acid (TNBS) and oxazolone, two haptenating agents, can induce bowel inflammation that is similar to CD and UC, respectively^15^,^16^,^17^. Previous studies have focused mostly on the ameliorating effect of VD on TNBS-induced colitis, a model of Th1-mediated inflammation^18^,^19^,^20^. Oxazolone-induced colitis, which mimicked the pathological process of UC, has received less attention. Considering the different immunopathology of the two diseases, the role of VD in UC needs a more detailed exploration.

In this study, mice with either TNBS-induced or oxazolone-induced colitis were treated with VD to compare the protective effects of VD on UC and CD. Our aim was to compare the effect of VD on Th1-induced and Th2-induced colitis and explore the mechanism. The results of this study may contribute to the selective usage of VD in the treatment of IBD.

## Results

### Vitamin D attenuated TNBS-induced colitis but not oxazolone-induced colitis

**Figure 1:**
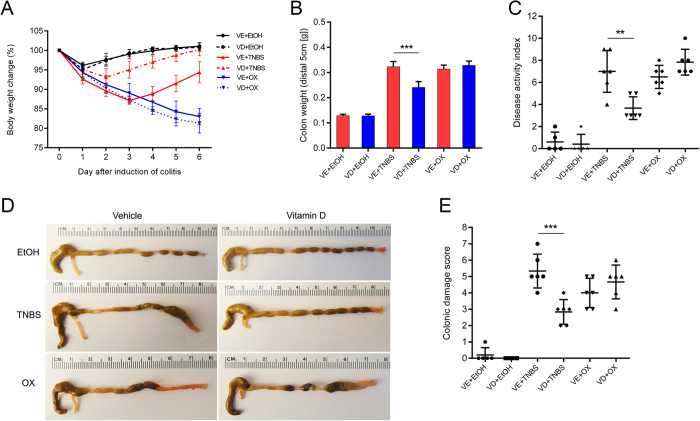
Effects of vitamin D treatment on the development of TNBS- and oxazolone-induced colitis. (**A**) Body weight changes (percentage of the original body weight) over time (days) in the VE and VD groups following 50% ethanol, TNBS or oxazolone treatment (n = 6–8 in each group). (**B**) Colon weight (distal 5 cm of each colon) in every experimental group. ***P < 0.001 (n = 5–6 in each group). (**C**) Disease activity index. *P < 0.01 (n = 5–6 in each group). (**D**) Typical gross morphology of the colon on day 4 from each treatment group. (**E**) Colonic damage score. ***P < 0.001 (n = 5–6 in each group). VE, vehicle treatment. VD, vitamin D treatment. TNBS, 2,4,6-trinitrobenzene sulphonic acid. OX, oxazolone. EtOH, 50% ethanol.

**Figure 2:**
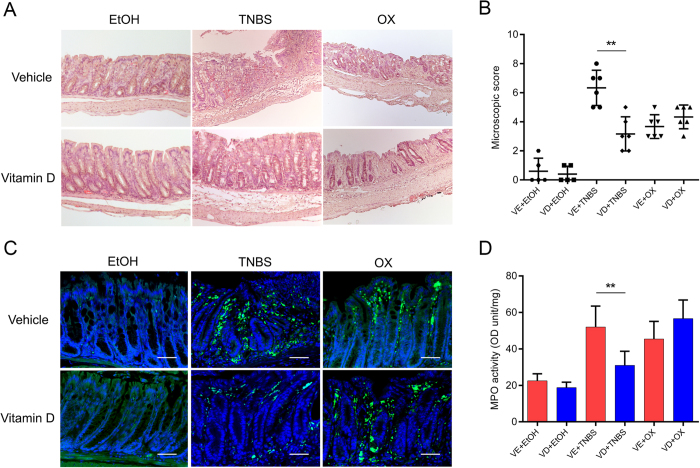
Vitamin D dramatically attenuates immune cell infiltration in TNBS-induced colitis and slightly aggravates infiltration in oxazolone-induced colitis. (**A**) Haematoxylin and eosin staining of colons on day 4 following different treatment methods. (**B**) Microscopic scoring of each colonic slide based on haematoxylin and eosin staining. **P < 0.01 (n = 5–6 in each group). (**C**) Immunofluorescence staining of colons with anti-CD4 antibody on day 2 after different treatments. Original magnification: 200×, bar = 100 μm. (**D**) MPO activity. **P < 0.01 (n = 5–6 in each group).

Both TNBS and oxazolone injected per rectum induced colitis successfully, as evidenced by body weight loss, rectal bleeding, colonic oedema and hyperaemia. VD treatment improved the overall status of TNBS mice but not that of oxazolone-treated mice. TNBS- or oxazolone-treated colons showed severe hyperaemia, inflammation and ulcerative necrosis, as well as an increased colonic damage score compared with vehicle-treated mice. VD rescued the above pathologic changes in TNBS mice but not in oxazolone-treated mice (Fig. 1). Transmural inflammation, characterized by inflammatory cell infiltration, colonic mucosal ulcerations, loss of goblet cells and fibrosis, could be observed in TNBS- and oxazolone-treated colons. The local structure was almost restored and largely remained intact in the VD+TNBS group. However, the tissue destruction was worsened in the VD+oxazolone group. Inflammatory cell infiltration was observed in TNBS- and oxazolone-treated colons. This infiltration was inhibited by VD treatment in the former, but not in the latter model (Fig. 2A,B). As shown by immunostaining, massive infiltration of CD4-positive cells was detected in the lamina propria of TNBS- and oxazolone-treated colons. CD4-positive cells were dramatically decreased in the VD+TNBS colons, but not in the VD+oxazolone colons (Fig. 2C). In addition, myeloperoxidase (MPO) activity was substantially reduced in VD+TNBS colonic mucosal lysates, whereas it was slightly increased in VD+oxazolone lysates; however, the difference was not significant (Fig. 2D). These findings suggest that VD may have a protective effect against TNBS-induced colitis but not oxazolone-induced colitis.

### Vitamin D suppressed intestinal apoptosis in TNBS-treated mice but not in oxazolone-treated mice

**Figure 3:**
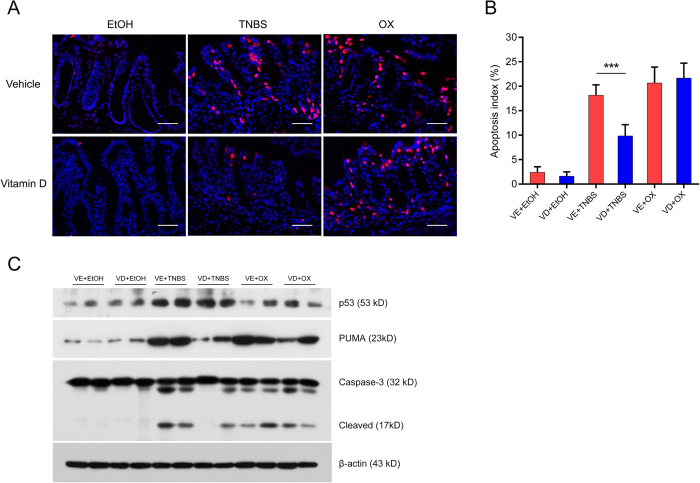
Vitamin D suppressed colonic cell apoptosis in TNBS-treated mice but not in oxazolone-treated mice. (**A**) Representative TUNEL staining of each treatment group on day 2. Red spots indicate TUNEL-positive apoptotic cells, Original magnification: 400×, bar = 50μm. (**B**) Apoptotic index in each group. The apoptotic index was defined as the percentage of TUNEL-positive crypts in 100 randomly chosen crypts in each colon slide. ***P < 0.001 (n = 5 in each group). (**C**) Western blot analyses of apoptotic proteins in different treatment groups on day 2. All electrophoresis was performed under the same condition, and gel images were cropped for concise presentation. The uncut images are provided in Supplementary Materials.

We next investigated whether the protective effect of VD on TNBS- or oxazolone-induced colitis was associated with its anti-apoptotic effect. We observed abundant apoptotic crypts after TNBS or oxazolone challenge by terminal deoxynucleotidyl transferase-mediated dUTP nick-end labelling (TUNEL) staining, whereas TUNEL-positive crypts were markedly reduced in VD+TNBS mice but not in VD+oxazolone mice (Fig. 3A,B). The increase in the expression of apoptotic proteins caused by TNBS administration was suppressed by VD treatment; however, in oxazolone-induced colitis, VD treatment appeared to have no effect (Fig. 3C). VD inhibited the apoptosis of colonic cells in a TNBS-induced colitis model but not in an oxazolone-induced colitis model.

### Vitamin D preserved tight junction function and decreased intestinal permeability in TNBS-treated mice but not in oxazolone-treated mice

**Figure 4:**
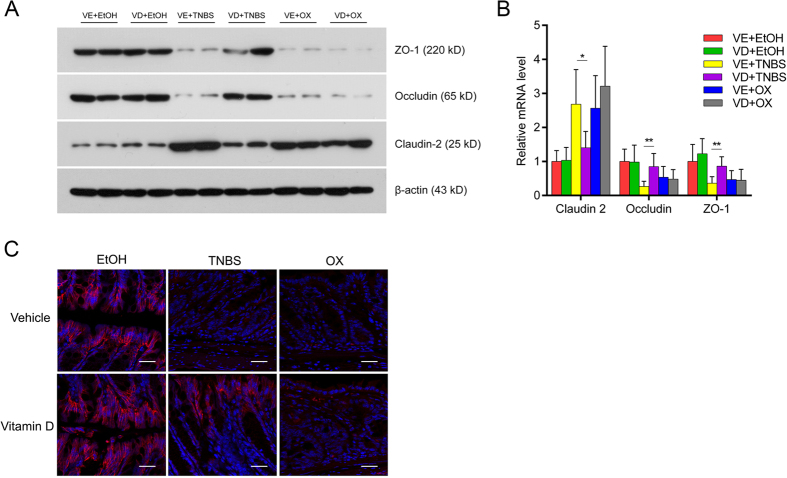
Vitamin D maintained tight junction function in TNBS-treated mice but not in oxazolone-treated mice. (**A**) Western blot analyses of the colonic mucosal levels of tight junction proteins in different treatment groups on day 2. (**B**) Relative mRNA expression of tight junction proteins in different treatment groups on day 2 by real-time PCR. *P < 0.05, **P < 0.01 versus vehicle treatment (n = 6 in each group). (**C**) Confocal microscopic images of occludin staining in different treatment groups on day 2. Original magnification: 800×, bar = 25 μm; red staining represents the intercellular expression of occludin, which were counterstained with DAPI (blue). All electrophoresis was performed under the same condition, and gel images were cropped for concise presentation. The uncut images are provided in Supplementary Materials.

Because the intestinal barrier function is influenced by colonic cell apoptosis and tight junction function, we also studied three typical tight junction proteins: occludin, zonula occludens-1 (ZO-1) and claudin-2. After the administration of TNBS and oxazolone, occludin and ZO-1 expression decreased significantly, demonstrating the impaired tight junction function. This decrease could be rescued by VD treatment in TNBS-induced colitis but not in oxazolone-induced colitis. The expression of claudin-2, a pore-forming protein, was elevated after the administration of TNBS and oxazolone, and this elevation was relieved by VD in the former model but not in the latter (Fig. 4A,B). TNBS and oxazolone administration resulted in a sparse, discontinuous expression of occludin in intestinal epithelial cells. VD could alleviate this change in the TNBS model but did not have an effect in the oxazolone model (Fig. 4C).

**Figure 5:**
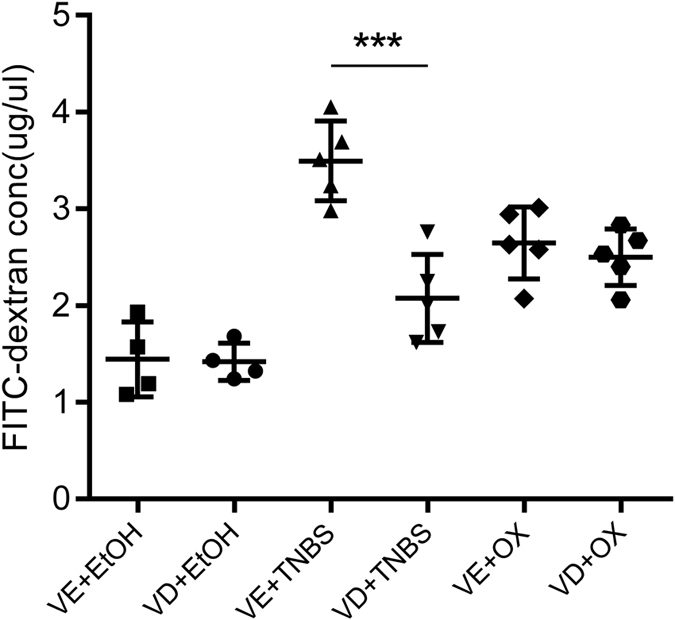
Vitamin D decreased the intestinal permeability in TNBS mice but not in oxazolone mice. Intestinal permeability to 4-kDa FITC-dextran was measured at 3 hours after gavage. (n = 4–5 in each group). ***P < 0.001 versus vehicle treatment.

Colonic cell apoptosis and disturbed tight junction function may lead to a disrupted intestinal barrier and increased gut permeability, as reflected by an increase in FITC-conjugated 4kD dextran (FD4) leaking from the lumen into the circulation. VD treatment partly alleviated intestinal FD4 leakage to the serum in TNBS-treated colons, but did not decrease intestinal permeability in oxazolone-treated colons (Fig. 5).

### Effects of vitamin D on T-cell function in TNBS-treated or oxazolone-treated mice

**Figure 6:**
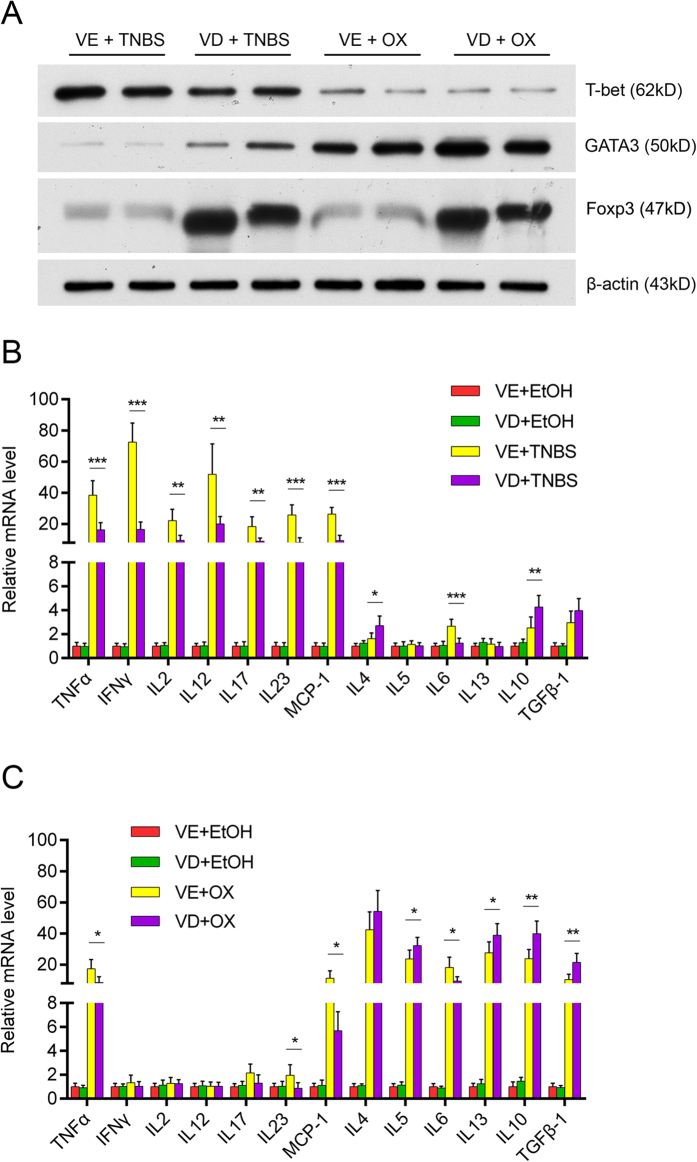
Effects of vitamin D on T-cell functions in TNBS- or oxazolone-treated mice. (**A**) Western blot analyses of the colonic mucosal levels of a Th1 transcription factor (T-bet), a Th2 transcription factor (GATA3) and a Treg cell mediator (Foxp3) in different treatment groups on day 2. (**B**) In TNBS-treated mice, vitamin D intervention suppresses all Th-1 mediated colonic inflammatory cytokines. **P < 0.01, ***P < 0.001 versus vehicle treatment (n = 6 in each group). (**C**) Vitamin D treatment slightly aggravates Th-2-mediated inflammatory cytokines induced by oxazolone and increases Treg-related cytokines. *P < 0.05, **P < 0.01 versus vehicle treatment (n = 6 in each group). All electrophoresis was performed under the same condition, and gel images were cropped for concise presentation. The uncut images are provided in Supplementary Materials.

We further investigated the mechanism of the different effect of VD on TNBS- and oxazolone-induced colitis. The observed reduction of the Th1 transcription factor T-bet was reduced substantially by VD treatment. The Th2 transcription factor GATA3 increased in oxazolone-treated colonic mucosa and was further augmented by VD treatment. Foxp3, the marker of regulatory T cells (Tregs), was enhanced in both TNBS- and oxazolone-induced colitis after VD treatment (Fig. 6A).

We also investigated the expression level of Th1-, Th2-, Th17- and Treg- related inflammatory mRNAs. We examined Th1-cytokines (TNF-α, IFN-γ, IL-2 and IL-12), Th2- cytokines (IL-4, IL-5, IL-6 and IL-13), Th17- cytokines (IL-17, IL-23), Treg- cytokines (IL-10, TGFβ-1) and chemokine (MCP-1). Most of the above cytokines increased following stimulation with TNBS. VD attenuated this tendency, with significance reached for all Th-1 and Th17-mediated cytokines (Fig. 6B). In the oxazolone-induced colitis model, the Th-2-mediated immune response was responsible for the main proinflammatory process, and VD increased the relative levels of Th2- and Treg- mediated cytokines (Fig. 6C).

## Discussion

This study demonstrated that VD could relieve TNBS-induced colitis by down-regulating Th1- and Th17-mediated inflammation. However, VD did not significantly ameliorate oxazolone-induced bowel inflammation, possibly due to the counteracting effect of enhanced Th2 and Treg cytokine secretion.

The relationship between VD/VDR and IBD has long been established^21^. VD deficiency is common, especially in CD patients, even when the disease is in remission^9^,^10^,^11^,^12^,^13^. VD supplementation might be helpful in the treatment and prevention of IBD^22^,^23^. A recent meta-analysis suggested an increased risk of both UC and CD associated with several VDR polymorphisms^14^. In animal studies, VDR deficiency increased the susceptibility of mice to TNBS-induced colitis, dextran sodium sulfate (DSS)-induced colitis, T-cell transfer-induced colitis and genetic models of IBD^24^,^25^. Enhanced intestinal epithelial VDR signalling inhibited experimental colitis models, including TNBS-induced colitis, DSS-induced colitis and T-cell transfer-induced colitis^18^. However, the literature has only limited evidence to verify the protective effect of VD on IBD. A systematic review published in 2012 only found four high-quality studies on this topic, two of which were actually comparing effects of different VD analogues^26^.

The divergence in the protective potential of VD might lie in differences between the two diseases. A positive effect of VD treatment was mostly observed in CD patients^22^,^23^, and common animal models of IBD, such as TNBS-induced and DSS-induced colitis, mimicked exclusively the pathogenesis of CD^19^,^20^,^24^,^25^. Clinical and experimental studies have paid limited attention to UC. In this study, we observed that VD treatment significantly reduced colonic cell apoptosis, preserved tight junction function and maintained the permeability of the epithelial barrier in TNBS colitis. However, we did not find these beneficial effects in oxazolone-induced colitis.

Because the mechanism of the suppressive role of VD on IBD had been associated with its immunoregulatory effects, the question arose of whether the different effects of VD treatment on CD and UC were associated with different immunological features of the two diseases. Clinical and animal studies have demonstrated that the major immunopathological feature of CD is an excessive Th1 and Th17 T cell response, characterized by increased IL-12, IFN-γ, TNF-α, IL-6, IL-17 and IL-23 production^2^,^3^,^4^,^5^. In murine models, TNBS-induced colitis was characterized by dense transmural inflammation, similar to that observed in CD, associated with a Th1 response dominated by IL-12, IFN-γ and TNF-α as well as a Th17 response with excessive IL-17 and IL-23^4^,^15^. In contrast, the main immunopathological feature of UC is increased IL-4 and/or IL-13 production^6^,^7^,^16^,^17^.Oxazolone-induced colitis exhibited relatively mild inflammation, including gut epithelial cell disruption and an activated Th2 cell response^16^,^17^. IL-4 increased in the early stage and was followed by IL-13^16^,^17^. IL-4 and IL-13 antibodies have been shown to effectively treat oxazolone-induced colitis and UC patients^27^,^28^.

VD can ameliorate TNBS-induced colonic inflammation by suppressing the Th1- and Th17-mediated inflammatory response and enhancing the secretion of anti-inflammatory cytokines by Treg cells, as observed in this study and in previous reports^19^,^20^. Meanwhile, we used oxazolone as a Th2-mediated colitis model in which VD could promote the expression of Th2-mediated proinflammatory cytokines and regulatory T cell-mediated anti-inflammatory cytokines simultaneously. Possibly due to the counteracting effects in this system, VD did not show significant protective effect on oxazolone-induced colitis. Because TNBS and oxazolone induce bowel inflammation with mechanisms similar to CD and UC, respectively, these results imply that VD may have divergent effects on CD and UC patients.

The immunoregulatory role of VD/VDR has been identified in a number of autoimmune diseases, including systemic lupus erythematosus, allograft rejection, autoimmune diabetes mellitus and experimental allergic encephalomyelitis. In the absence of VDR, Th1 cell-driven IBD was more severe, while Th2 cell-driven asthma did not develop^25^,^29^. Calcitriol has been shown to be a prominent negative regulator of Th1-mediated immune responses, whereas it has no effect or even an augmenting effect on Th2 responses^20^. 1,25(OH)_2_D_3_ can inhibit the production of TNF-α, IL-17 and IFN-γ and stimulate the expression of IL-4, IL-5 and IL-10 by CD4 positive T cells^30^. It can also enhance the development of IL-10–producing CD4 positive T cells isolated from patients with multiple sclerosis and CD^31^. In oxazolone-induced, Th2-mediated colitis, VD enhanced both Th2- and Treg-mediated inflammation, and the up-regulated Treg cells could secrete anti-inflammatory cytokines, such as IL-10 and TGF-β1, that could partially counteract Th2 inflammatory response. The balance of the two counteracting effects made the effect of VD on oxazolone-induced colitis insignificant.

Oxazolone-induced colitis is characterized by an early increase in IL-4 followed by an increase in IL-13^16^,^17^. The cellular origin of IL-4 and IL-13 has been associated with natural killer T (NKT) cells^32^. NKT cells are a special group of cells with both the T cell receptor and NK cell receptors on the cell surface^32^. The depletion of cells bearing a marker found on NKT cells extracted from UC tissue, followed by stimulation and culture *in vitro*, could lead to a substantial decrease in IL-13 production^6^. NKT cells express VDR, and invariant NKT cells from VDR KO mice are intrinsically defective and lack T-bet expression^33^,^34^. By augmenting the VDR function in invariant NKT cells using VD supplementation, IL-4 and IL-13 secretion could be up-regulated and therefore aggravate Th2-mediated colitis. Our future studies will aim to investigate how VD/VDR could influence the function of invariant NKT cells.

In conclusion, this study suggests that VD treatment can attenuate TNBS-induced Th1 mediated colitis, but has no effect on oxazolone-induced Th2-mediated colitis. The absence of a protective effect was due to the simultaneous increase in Th2 and Treg cytokine secretion, which counteracted each other. Our study helps elucidate the different effects of VD on CD and UC patients. We hope that the results of this study will accelerate the preparation of a large prospective study on VD treatment for the two types of IBD.

## Materials and Methods

### Animals

This study was approved by the Institutional Ethical Committee of Shengjing Hospital, China Medical University. All the procedures were performed in accordance with the approved guidelines. Male and female adult C57BL/6J mice were purchased from the Centre for Experimental Animals of China Medical University. The mice were 8-10 weeks old, and the female: male ratio was 1:1 in each group. The animals weighed 20–25 g. All mice were kept in specific pathogen-free static cages with a 12-h light/dark cycle. Chow pellets and tap water were available ad libitum.

### Induction of colitis

The mice were anesthetized by injecting a cocktail of xylazine (Rompun 2%; Bayer AG, Leverkusen, Germany) and ketamine (Ketavest; 100 mg/ml; Pfizer, Inc., New York, NY, USA) intraperitoneally (i.p.). TNBS was prepared by dissolving 5% TNBS (Sigma, St. Louis, MO, USA) in an equal volume of absolute ethanol to obtain a working solution of 2.5% TNBS in 50% ethanol. Oxazolone was dissolved in 50% ethanol to obtain a 5% concentration. To induce colitis, the mice were administered a dose of 100 mg/kg (4 μl/g body weight) TNBS or 5 μl/g body weight of 5% oxazolone per rectum with an 18-gauge stainless steel gavage needle. The control group was given the same volume of 50% ethanol without TNBS or oxazolone.

### Vitamin D treatment

The TNBS, oxazolone and vehicle groups were randomly divided into two subgroups. One subgroup was treated with a VD analogue, paricalcitol (Sigma), dissolved in 90:10 propylene glycol: ethanol at 0.5 μg/kg body weight, while the vehicle subgroups were given the solvent only. Paricalcitol or vehicle was given through i.p. injection 30 min prior to the induction of colitis, and at 1, 3 and 5 days after the induction of colitis.

### Evaluation of colonic damage

The mice were sacrificed by cervical dislocation on day 4 after TNBS or oxazolone injection. The presence or absence of diarrhoea was observed and recorded before sacrifice. When dissecting the colon, the presence or absence of adhesion was noted. The entire colon was harvested and observed thoroughly for gross examination. We imaged the colon morphology and weighed a 5-cm length of the distal colon. Then, the colon was cut open longitudinally, washed with water and extended on a plastic block for the observation of ulceration. The colonic damage score was calculated according to a macroscopic scoring system^35^. The disease activity index was calculated for each animal on the basis of stool consistency, rectal bleeding and weight loss percentage^36^.

### Selection of time points

In our preliminary studies, we found that clinical symptoms were most severe on day 2 after TNBS administration, when the mice showed the most body weight loss and the least vitality. Moreover, the difference was most significant between the VD treatment group and the control group on day 2. Thus, on day 2, we evaluated CD4 cell infiltration, apoptosis of the epithelial cells and the function of tight junctions by performing TUNEL staining, immunofluorescence staining for tight junction proteins, permeability measurement, MPO activity tests, western blotting for T-cell transcription factors and real-time PCR for inflammatory cytokines. The local damage of the colon was the most severe on day 4 after colitis induction, and after day 4, the local structures started to recover. Thus, on day 4, we performed gross observation of the colon and haematoxylin&eosin staining of the sections to investigate structural damage of the colon.

### Histology

The distal colons were harvested on day 4 after colitis induction and fixed overnight with 4% formaldehyde in phosphate buffer saline (PBS, pH = 7.4), dehydrated with graded alcohol, placed in xylene and embedded in paraffin. Sections (4 μm) were stained with H&E (Beyotime Institute of Biotechnology, Haimen, China) at room temperature. Five areas were randomly chosen in each section and examined at 100× magnification. In each field, colon microscopic scoring was performed independently by two pathologists who were blinded to the study design; the scoring was performed according to a microscopic scoring system^37^. To analyze the infiltration of inflammatory cells or the structure of the tight junctions, the sections were incubated with anti-CD4 (1:200 dilution; Santa Cruz Biotechnology, Inc., Dallas, TX, USA) or anti-occludin (1:200 dilution; Invitrogen; Thermo Fisher Scientific, Waltham, MA, USA) antibodies, followed by secondary antibodies conjugated with AlexaFluor 488 (1:1,500 dilution) or AlexaFluor 555 (1:1,500 dilution) from Invitrogen. The immunostained antigens were visualized using a Leica DFC425 fluorescence microscope [Leica Microsystems (Schweiz) AG, Heerbrugg, Switzerland] and occludin staining was observed using a confocal laser-scanning microscope (C1, Nikon, Japan).

### Myeloperoxidase (MPO) activity

MPO was expressed in units per milligram of distal colon tissue and analyzed using a MPO assay kit according to manufacturer’s instructions (CytoStore, Alberta, Canada), as previously reported^37^.

### Western blot

We sacrificed the mice on day 2 after TNBS or oxazolone treatment and harvested the distal colonic mucosal lysates. The lysates were separated by polyacrylamide gel electrophoresis, and the proteins were transferred electrophoretically onto polyvinylidene difluoride membranes (EMD Millipore, Billerica, MA, USA). Then the membranes were incubated with primary antibodies. The following primary antibodies were used in this study: anti-β-actin (1:2,000 dilution; Santa Cruz); anti-p53 (1:2,000 dilution), anti-p53 unregulated modulator of apoptosis (PUMA; 1:2,000 dilution), anti-caspase 3 (1:1,000 dilution), anti-T-bet (1:1000 dilution), anti-GATA3 (1:1000 dilution), and anti-Foxp3 (1:1000 dilution) antibodies (all from Cell Signaling Technology, Danvers MA, USA); and anti-zonula occludens-1 (ZO-1; 1:2,000 dilution), anti-occludin (1:2,000 dilution) and anti-claudin-2 (1:2000 dilution) antibodies (all from Invitrogen).

### Real-time PCR

The mice were sacrificed on day 2 after TNBS or oxazolone treatment. We harvested a piece of distal colon approximately 1.0 cm in length from the same segment in all the mice. Colonic mucosa was isolated by careful scraping and RNA was isolated from the colonic mucosa with Trizol (Invitrogen). First-strand cDNAs were synthesized from 2 μg of total RNA in a 20-μl reaction system with M-MLV reverse transcriptase (Invitrogen) and random primers. Real-time PCR was performed using a Bio-RAD IQ5 real-time system and SYBR green PCR Master Mix (Takara Biotechnology Co., Japan). The relative transcription levels of the mRNAs were calculated according to the 2^−ΔΔCt^ formula. β-2 microglobulin (B2M) was used as an internal control. Mouse real-time PCR primers are shown in Table 1.

### TUNEL staining

Sections of the distal colons were used. Terminal deoxynucleotidyl transferase-mediated dUTP nick-end labelling (TUNEL) staining was performed to detect intestinal cell apoptosis using an *In Situ* Cell Death Detection Kit, TMR red (Roche Diagnostics, Indianapolis, IN, USA) according to the manufacturer’s instructions. The apoptotic index was defined as the percentage of TUNEL-positive-cell-containing crypts in 100 randomly chosen crypts in each colon slide.

### Intestinal permeability measurement

The mice were denied access to food but were allowed water for 4 hours before gavage. FITC-conjugated 4-kD dextran (FD4) from Sigma (50 mg/ml) was administered via gavage at 4 μl/g body weight through an 18-gauge stainless steel gavage needle. Blood serum was collected 3 hours later. Two hundred microliters of sample per well were added to a 96-well plate; then, the serum concentration of FD4 was measured using a Synergy HT plate reader (BioTek Laboratories, Inc., WA, USA), as previously described^38^,^39^.

### Statistical analyses

All continuous data are presented as the mean ± standard deviation (SD). Statistical comparison of continuous variables between groups was performed using Student’s t test or one-way ANOVA (followed by Games-Howell test) with GraphPad Prism software 6.0 (GraphPad Software Inc., La Jolla, CA, USA) and SPSS 17.0 (SPSS Inc., Chicago, IL, USA). Rank data were analyzed using a Wilcoxon rank test. *P* < 0.05 was considered significant.

## Additional Information

**How to cite this article**: Liu, T. *et al*. Vitamin D treatment attenuates 2,4,6-trinitrobenzene sulphonic acid (TNBS)-induced colitis but not oxazolone-induced colitis. *Sci. Rep.* **6**, 32889; doi: 10.1038/srep32889 (2016).

## Supplementary Material

Supplementary Information

## Figures and Tables

**Table 1:** Mouse-specific primer sequences used for real-time PCR.

Primer name	Forward (5′ - 3′)	Reverse (3′ - 5′)
mouse TNF-α	ATGAGCACAGAAAGCATGA	AGTAGACAGAAGAGCGTGGT
mouse IFN-γ	TTCTTCAGCAACAGCAAGGC	TCAGCAGCGACTCCTTTTCC
mouse IL-2	TTGTGCTCCTTGTCAACAGC	CTGGGGAGTTTCAGGTTCCT
mouse IL-4	AACGAGGTCACAGGAGAAGG	TCTGCAGCTCCATGAGAACA
mouse IL-5	ACCGAGCTCTGTTGACAAG	TCCTCGCCACACTTCTCTTT
mouse IL-6	CCTCTGGTCTTCTGGAGTACC	ACTCCTTCTGTGACTCCAGC
mouse IL-10	ATAACTGCACCCACTTCCCA	GGGCATCACTTCTACCAGGT
mouse IL-12	GATGACATGGTGAAGACGGC	AGGCACAGGGTCATCATCAA
mouse IL-13	GCAGCATGGTATGGAGTGTG	TGGCGAAACAGTTGCTTTGT
mouse IL-17	TCTCCACCGCAATGAAGACC	CACACCCACCAGCATCTTCT
mouse IL-23	GCTGTGCCTAGGAGTAGCAG	TGGCTGTTGTCCTTGAGTCC
mouse MCP-1	GCTCAGCCAGATGCAGTTAA	TCTTGAGCTTGGTGACAAAAACT
mouse TGF-β1	CCTGCAAGACCATCGACATG	TGTTGTACAAAGCGAGCACC
mouse Claudin-2	TATGTTGGTGCCAGCATTGT	TCATGCCCACCACAGAGATA
mouse Occludin	CCTCCAATGGCAAAGTGAAT	CTCCCCACCTGTCGTGTAGT
mouse ZO-1	CCACCTCTGTCCAGCTCTTC	CACCGGAGTGATGGTTTTCT
mouse B2M	CGGCCTGTATGCTATCCAGA	GGGTGAATTCAGTGTGAGCC

## References

[b1] Abraham C. & Cho J. H. Inflammatory bowel disease. N Engl J Med 361, 2066–78 (2009). 19923578 10.1056/NEJMra0804647 PMC3491806

[b2] Fuss I. J. . Disparate CD4 lamina propria (LP) lymphokine secretion profiles in inflammatory bowel disease. Crohn’s disease LP cells manifest increased secretion of IFN-γ, whereas ulcerative colitis LP cells increased secretion of IL-5. J Immunol. 157, 1261–70 (1996). 8757634

[b3] Monteleone G. . Interleukin 12 is expressed and actively released by Crohn’s disease intestinal lamina propria mononuclear cells. Gastroenterology 112, 1169–78 (1997). 9098000 10.1016/s0016-5085(97)70128-8

[b4] Sarra M., Pallone F., Macdonald T. T. & Monteleone G. IL-23/IL-17 axis in IBD. Inflamm Bowel Dis. 16, 1808–13 (2010). 20222127 10.1002/ibd.21248

[b5] Yen D. . IL-23 is essential for T cell mediated colitis and promotes inflammation via IL-17 and IL-6. J Clin Invest. 116, 1310–6 (2006). 16670770 10.1172/JCI21404 PMC1451201

[b6] Fuss I. J. . Non classical CD1d-restricted NK T cells that produce IL-13 characterize an atypical Th2 response in ulcerative colitis. J Clin Invest. 113, 1490–7 (2004). 15146247 10.1172/JCI19836 PMC406524

[b7] Heller F. . Interleukine-13 is the key effector Th2 cytokine in ulcerative colitis that affects epithelial tight junctions, apoptosis, and cell restitution. Gastroenterology 129, 550–64 (2005). 16083712 10.1016/j.gastro.2005.05.002

[b8] Cantorna M. T. & Mahon B. D. Mounting evidence for vitamin D as an environmental factor affecting autoimmune disease prevalence. Exp Biol Med. 229, 1136–42 (2004). 10.1177/153537020422901108 15564440

[b9] Cantorna M. T., McDaniel K., Bora S., Chen J. & James J. Vitamin D, immune regulation, the microbiota, and inflammatory bowel disease. Exp Biol Med. 239, 1524–30 (2014). 10.1177/1535370214523890 PMC4176535 24668555

[b10] Tan B. . Vitamin D Levels and bone metabolism in Chinese adult patients with inflammatory bowel disease. J Dig Dis. 15, 116–23 (2014). 24354597 10.1111/1751-2980.12118

[b11] Levin A. D. . Vitamin D deficiency in children with inflammatory bowel disease. Dig Dis Sci. 56, 830–6 (2011). 21222159 10.1007/s10620-010-1544-3

[b12] Ananthakrishnan A. N. . Higher predicted vitamin D status is associated with reduced risk of Crohn’s disease. Gastroenterology. 142, 482–9 (2012). 22155183 10.1053/j.gastro.2011.11.040 PMC3367959

[b13] Harries A. D. . Vitamin D status in Crohn’s disease: association with nutrition and disease activity. Gut. 26, 1197–203 (1985). 3877663 10.1136/gut.26.11.1197 PMC1432925

[b14] Xue L. N. . Associations between vitamin D receptor polymorphisms and susceptibility to ulcerative colitis and Crohn’s disease: a meta-analysis. Inflamm Bowel Dis. 19, 54–60 (2013). 22467262 10.1002/ibd.22966

[b15] Neurath M. F., Fuss I., Kelsall B. L., Stüber E. & Strober W. Antibodies to interleukin-12 abrogate established experimental colitis in mice. J Exp Med. 182, 1281–90 (1995). 7595199 10.1084/jem.182.5.1281 PMC2192205

[b16] Boirivant M., Fuss I. J., Chu A. & Strober W. Oxazolone colitis: a murine model of T helper cell type 2 colitis treatable with antibodies to interleukin 4. J Exp Med. 188, 1929–39 (1998). 9815270 10.1084/jem.188.10.1929 PMC2212414

[b17] Heller F., Fuss I. J., Nieuwenhuis E. E., Blumberg R. S. & Strober W. Oxazolone colitis, a Th2 colitis model resembling ulcerative colitis, is mediated by IL-13-producing NK-T cells. Immunity. 17, 629–38 (2002). 12433369 10.1016/s1074-7613(02)00453-3

[b18] Liu W. . Intestinal epithelial vitamin D receptor signaling inhibits experimental colitis. J Clin Invest. 123, 3983–96 (2013). 23945234 10.1172/JCI65842 PMC3754241

[b19] Daniel C. . The new low calcemic vitamin D analog 22-ene-25-oxa-vitamin D prominently ameliorates T helper cell type 1-mediated colitis in mice. J Pharmacol Exp Ther. 319, 622–31 (2006). 16914561 10.1124/jpet.106.107599

[b20] Daniel C., Sartory N. A., Zahn N., Radeke H. H. & Stein J. M. Immune modulatory treatment of trinitrobenzene sulfonic acid colitis with calcitriol is associated with a change of a T helper (Th) 1/Th17 to a Th2 and regulatory T cell profile. J Pharmacol Exp Ther. 324, 23–33 (2008). 17911375 10.1124/jpet.107.127209

[b21] Mouli V. P. & Ananthakrishnan A. N. Review article: vitamin D and inflammatory bowel diseases. Aliment Pharmacol Ther. 39, 125–36 (2014). 24236989 10.1111/apt.12553 PMC3872479

[b22] Jørgensen S. P. . Clinical trial: vitamin D3 treatment in Crohn’s disease – a randomized double-blind placebo controlled study. Aliment Pharmacol Ther. 32, 377–83 (2010). 20491740 10.1111/j.1365-2036.2010.04355.x

[b23] Yang L. . Therapeutic effect of vitamin D supplementation in a pilot study of Crohn’s patients. Clin Transl Gastroenterol. 4, e33 (2013). 23594800 10.1038/ctg.2013.1 PMC3636524

[b24] Froicu M. & Cantorna M. T. Vitamin D and the vitamin D receptor are critical for control of the innate immune response to colonic injury. BMC Immunol. 8, 5–15 (2007). 17397543 10.1186/1471-2172-8-5 PMC1852118

[b25] Froicu M. . A crucial role for the vitamin D receptor in experimental inflammatory bowel diseases. Mol Endocrinol. 17, 2386–92 (2003). 14500760 10.1210/me.2003-0281

[b26] Nicholson I., Dalzell A. M. & El-Matary W. Vitamin D as a therapy for colitis: a systematic review. J Crohns Colitis 6, 405–11 (2012). 22398085 10.1016/j.crohns.2012.01.007

[b27] Kasaian M. T. . Therapeutic activity of an interleukin-4/interleukin-13 dual antagonist on oxazolone-induced colitis in mice. Immunology 143, 416–27 (2014). 24831554 10.1111/imm.12319 PMC4212955

[b28] Jovani M., Fiorino G. & Danese S. Anti-IL-13 in inflammatory bowel disease: from the bench to the bedside. Curr Drug Targets 14, 1444–52 (2013). 23746199 10.2174/13894501113149990170

[b29] Wittke A., Weaver V., Mahon B. D., August A. & Cantorna M. T. Vitamin D receptor-deficient mice fail to develop experimental allergic asthma. J Immunol. 173, 3432–6 (2004). 15322208 10.4049/jimmunol.173.5.3432

[b30] Borgogni E. . Elocalcitol inhibits inflammatory responses in human thyroid cells and T cells. Endocrinology. 149, 3626–34 (2008). 18372324 10.1210/en.2008-0078

[b31] Correale J., Ysrraelit M. C. & Gaitán M. I. Immunomodulatory effects of Vitamin D in multiple sclerosis. Brain. 132, 1146–60 (2009). 19321461 10.1093/brain/awp033

[b32] Akbari O. . Essential role of NKT cells producing IL-4 and IL-13 in the development of allergen-induced airway hyperreactivity. Nat Med. 9, 582–8 (2003). 12669034 10.1038/nm851

[b33] Cantorna M. T., Zhao J. & Yang L. Vitamin D, invariant natural killer T-cells and experimental autoimmune disease. Proc Nutr Soc. 71, 62–6 (2012). 21996367 10.1017/S0029665111003193 PMC3733090

[b34] Yu S. & Cantorna M. T. The vitamin D receptor is required for iNKT cell development. Proc Natl Acad Sci USA 105, 5207–12 (2008). 18364394 10.1073/pnas.0711558105 PMC2278204

[b35] Butzner J. D., Parmar R., Bell C. J. & Dalal V. Butyrate enema therapy stimulates mucosal repair in experimental colitis in the rat. Gut. 38, 568–73 (1996). 8707089 10.1136/gut.38.4.568 PMC1383116

[b36] Cooper H. S., Murthy S. N., Shah R. S. & Sedergran D. J. Clinicopathologic study of dextran sulfate sodium experimental murine colitis. Lab Invest. 69, 238–49 (1993). 8350599

[b37] Appleyard C. B. & Wallace J. L. Reactivation of hapten-induced colitis and its prevention by anti-inflammatory drugs. Am J Physiol Gastrointest Liver Physiol. 269, G119–25 (1995). 10.1152/ajpgi.1995.269.1.G119 7631788

[b38] Du J. . 1,25-Dihydroxyvitamin D Protects Intestinal Epithelial Barrier by Regulating the Myosin Light Chain Kinase Signaling Pathway. Inflamm Bowel Dis. 21, 2495–506 (2015). 26287999 10.1097/MIB.0000000000000526 PMC4646414

[b39] Su L. . TNFR2 activates MLCK-dependent tight junction dysregulation to cause apoptosis-mediated barrier loss and experimental colitis. Gastroenterology 145, 407–15 (2013). 23619146 10.1053/j.gastro.2013.04.011 PMC3722284

