# Supplementary material for: Vitamin D treatment attenuates 2,4,6-trinitrobenzene sulphonic acid (TNBS)-induced colitis but not oxazolone-induced colitis
Source: Sci Rep. 2016 Sep 13;6:32889. doi: 10.1038/srep32889 (PMC5020649; doi:10.1038/srep32889)

Vitamin D treatment attenuates 2,4,6-trinitrobenzene sulphonic acid (TNBS)-induced colitis but not oxazolone-induced colitis

Tianjing Liu, Yongyan Shi, Jie Du, Xin Ge, Xu Teng, Lu Liu, Enbo Wang and Qun Zhao

Pathogens excluded in SPF

According to the laboratory animal-Microbiological standards and monitoring instructions (GB 14922.2-2011), the following organisms were excluded under SPF status in our lab:

Salmonella app.
Yersinia pseudotuberculosis
Yersinia enterocolitica
Pathogenic dermal fungi
Streptobacillus moniliformis
Bordetella bronchiseptica
Mycoplasma spp.
Corynebacterium kutscheri
Tyzzer's organism
O115 a, C,K(B)*Escherichia coli* O115 a, C,K(B)
Pasteurella pneumotropica
Klebsiella pneumoniae
Staphylococcus aureus
β-hemolyticstreptococcus
Pseudomonas aeruginosa

Uncut gels

Fig 3C

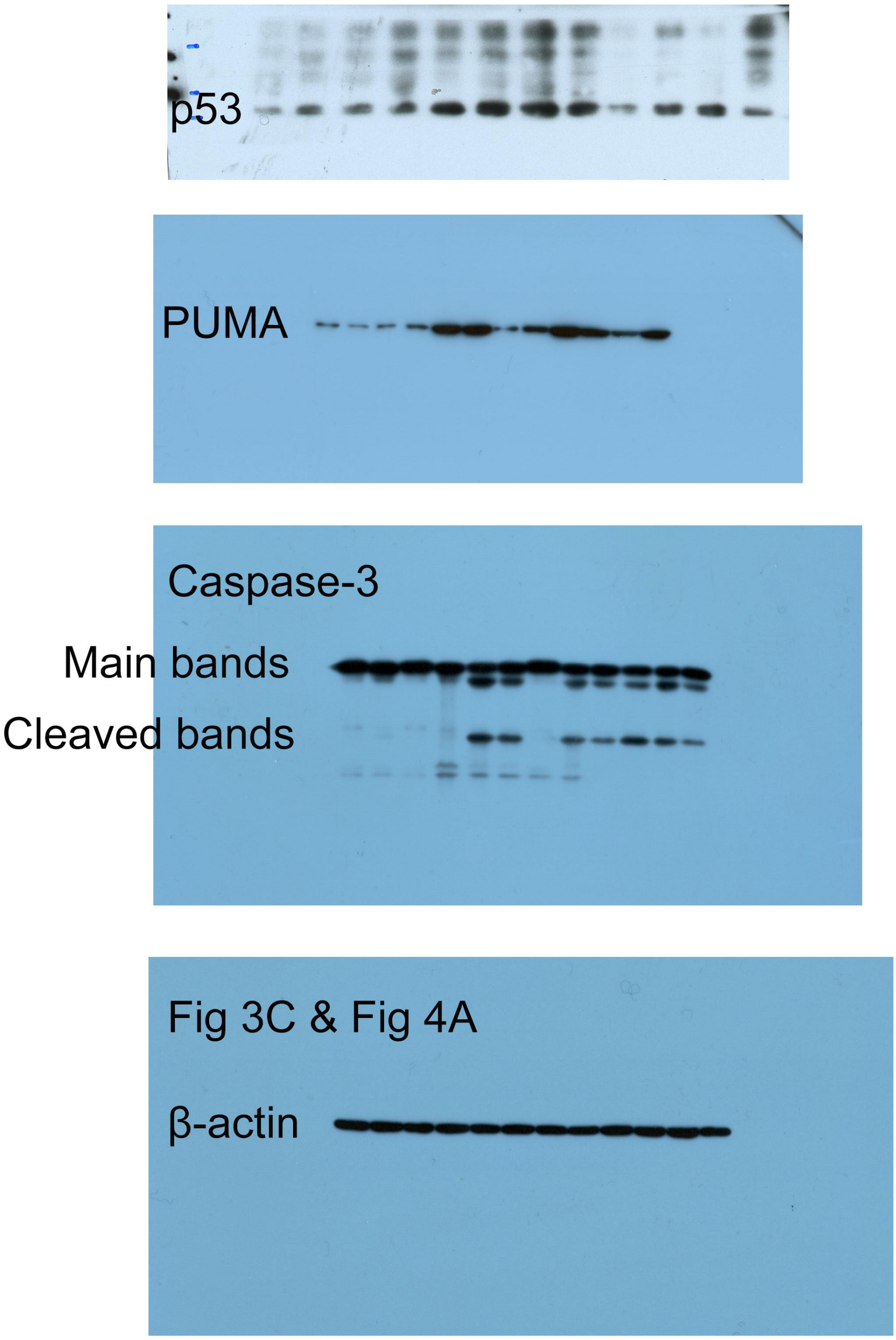

Fig 4A

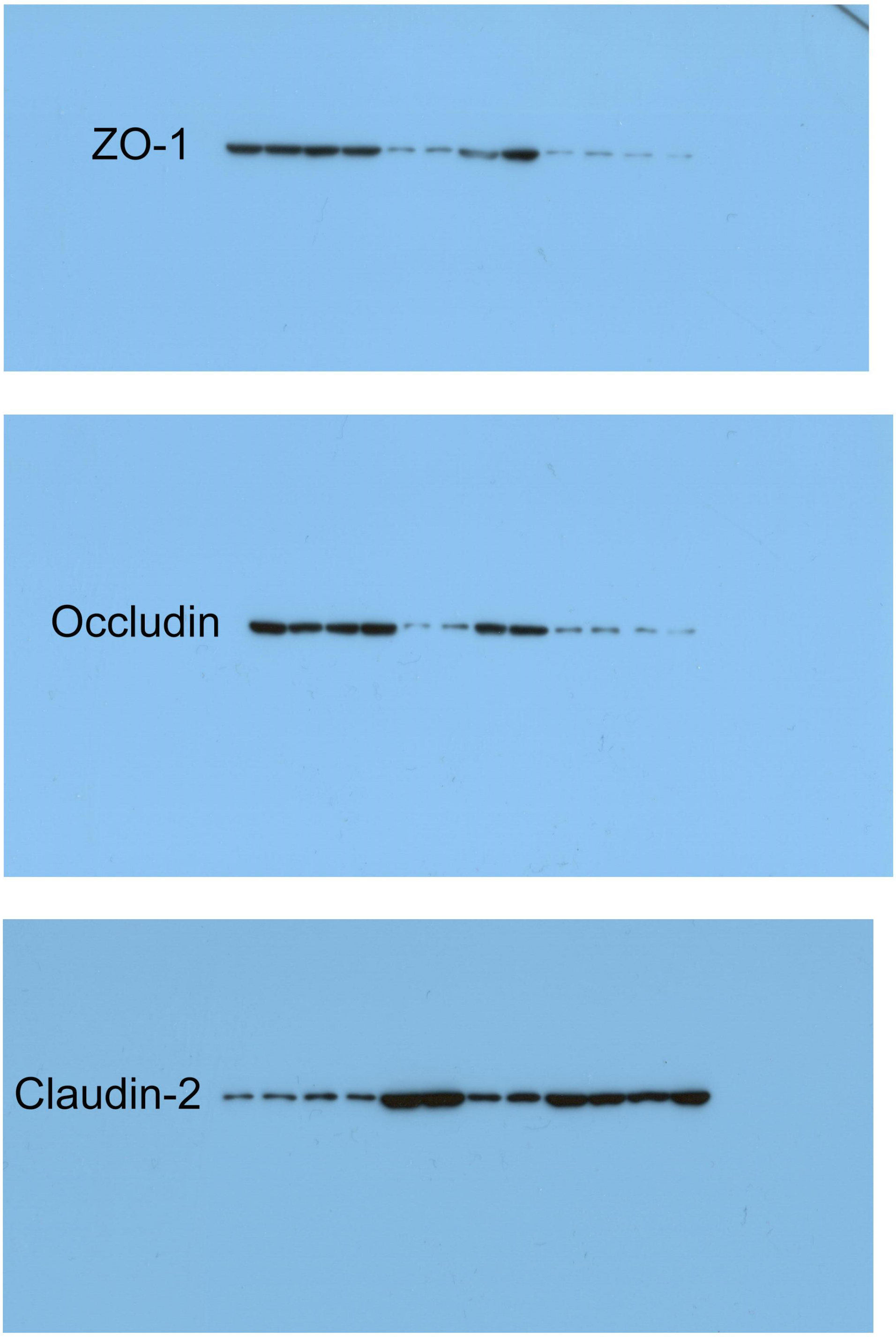

Fig 6A

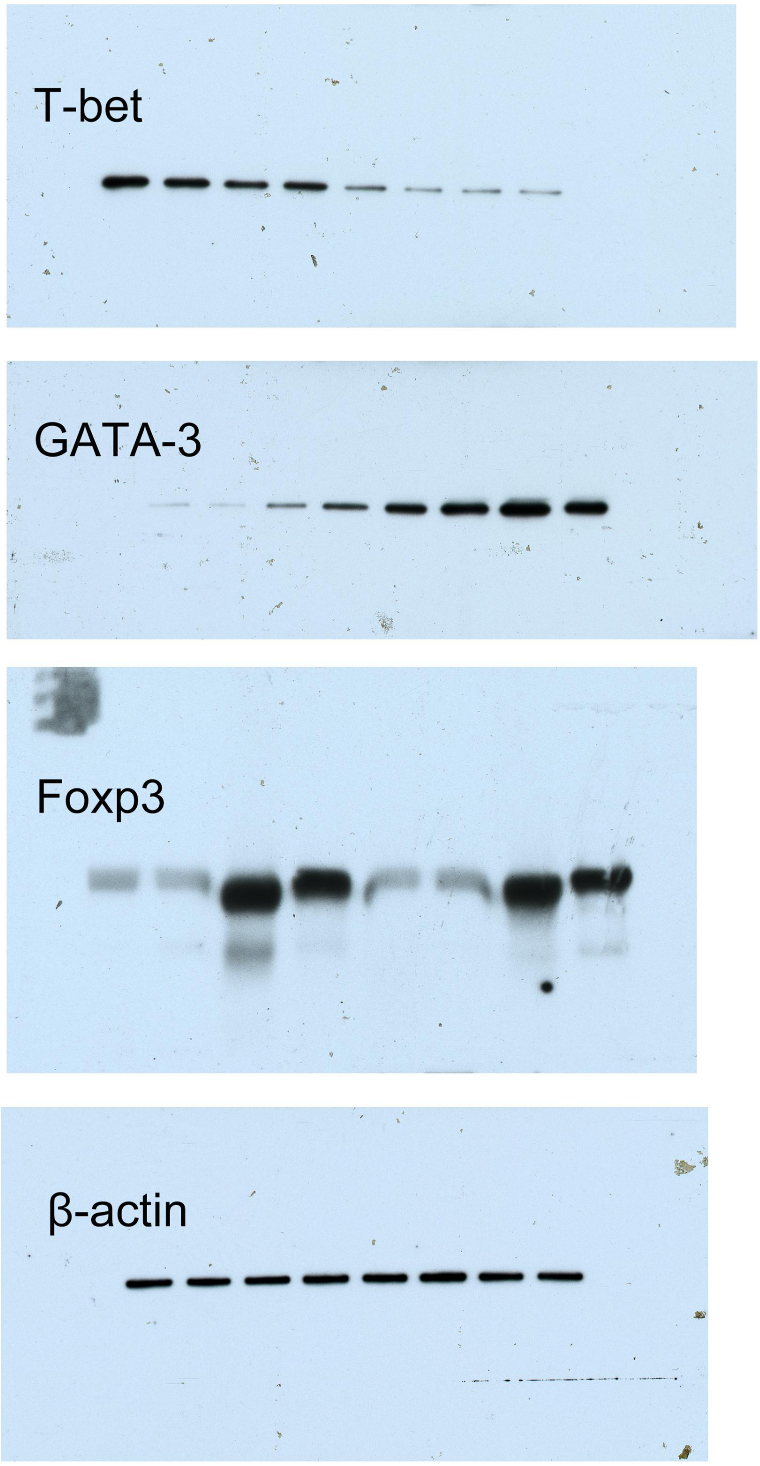

Supplement: Supplementary Information [file srep32889-s1.pdf]
